# Supplementary material for: Does LSD confer lasting psychological resilience? an investigation of naturalistic users experiencing job loss
Source: PLoS One. 2024 Jun 20;19(6):e0304991. doi: 10.1371/journal.pone.0304991 (PMC11189191; doi:10.1371/journal.pone.0304991)
Supplement: Attachment — Submitted filename: Response_to_Reviewers.docx [file pone.0304991.s001.docx]

Review Comments to the Author

Reviewer #1: This is an interesting and important study. In short, the article is well constructed and well written.

Author response: Thank you for your kind words and I’m happy to hear that you found the study interesting and important.

I have a minor suggestion. The methodology section describes "Survey respondents were randomly selected from across the U.S. and compensated $30 for participating."

According to me, this make slight confusion with the statement "Using purposeful respondent exclusion criteria, about purposeful exclusion criteria". Is this necessary to highlight the temporal precedence? Cause every study has some exclusion criteria with the logic. Can the statement be modified in any other format?

Did the compensation of $30 for participating hamper the authenticity of the study? I think, a statement regarding this issue would be better.

Author Response: Thank you for bringing this lack of clarity regarding the exclusion criteria applied in the analyses to my attention. I can see why my sole reference to the “purposeful exclusion criteria” in the original submissions’ abstract was unclear and needed modifying. To be clear, because the study data draw on a cross-sectional survey panel, purposeful exclusion criteria are indeed necessary to establish temporal precedence among the variables in my study (i.e., LSD use and psychological distress as shown graphically in Fig 1).

It is true that every study has some type of exclusion criteria. However, what I now try to highlight in my revised Abstract and Method section (pages 2 & 7) is that by excluding data on unemployed individuals who used LSD since their unemployment began, I can better explore whether LSD use prior to their job loss can predict psychological distress following their job loss. I hope my minor edits have helped clarify this in my revision.

Thank you as well for bringing your concerns regarding the payment of participants to my attention. It is important to understand that the NSDUH survey is a long survey with a large array of questions regarding individuals’ drug use, health, and general personal situation. Although I was not involved in the data collection (my manuscript makes secondary use of the data), I assume that the $30 compensation was necessary to incentivize enough respondents to participate. That being said, I do not think compensation for participation reduced the authenticity of the data (and as a result my findings) because, as reported in the article, participants were randomly sampled across the United States. This means that financially-motivated participants with little desire for answering the survey questions honestly could not self-select into the study (as would be the case for studies collecting data via online platforms like MTurk or Prolific). Furthermore, considering that participants would be unaware of my particular interest in testing the link between LSD use and psychological distress following job loss, it is unclear to me how their response patterns would explain the current study’s findings. Although this might be the case for experimental studies using overly salient experimental conditions, my findings make secondary use of a large, openly available dataset. Please let me know if you have additional concerns regarding this point.

Reviewer #2: The manuscript is technically sound with conclusions clearly demonstrable from the data presented, the statistical analysis is rigorous and appropriate, the data is from a publicly available dataset, and is written in fully intelligible standard English.

Author Response: Thank you very much.
